# On Lithia

**Published:** 1871

**Authors:** 


### Article 130.—*On Lithia.*

[Medical Times and Gazette, December 3.]

*Lithia* and its salts were introduced into the British Pharmacopœia of 1864. More than thirty years ago Mr. Ure and some other authorities called attention to the remarkable solvent powers which the carbonate of lithia possessed over uric acid calculi; powers which much exceed those possessed by the other alkaline carbonates, and Mr. Ure suggested the injection of solutions of the carbonate of lithia into the bladder, with the object of dissolving calculi formed wholly or in part of uric acid. But it is to Dr. Garrod that we owe the introduction of the lithia salts into medical practice. Extending the experiments of Mr. Ure, &c., he found that the carbonate of lithia could completely remove gouty deposits of urate of soda from cartilages incrusted by them, while carbonate of potash acted less strongly on them, and carbonate of soda left them unaltered. This encouraged him to make a trial of the lithia salts clinically, and with highly satisfactory results. He finds them of great value for keeping uric acid in solution during its passage through the urinary organs, and for preventing its deposition in the structures of the body; it seems, also, that they may be of service in removing gouty concretions when formed. The carbonate is a much more powerful diuretic than the salts of potash or soda, and may be given with great advantage, as a prophylactic, in chronic gout, calculus, &c. Its dose is from three to six grains, and is best given in a state of free dilution. The value of the lithia salts, especially the carbonate, has been widely recognized. The lithia spring of Baden* have gained a considerable reputation, and Prof. Roscoe has found lithium in the thermal waters of Bath. Dr. Garrod has stated that he has known a few, but a very few, instances "in which the long-continued use of the drug has appeared to cause symptoms referable to the nervous system, as shaking or trembling of one hand, which has disappeared on the omission of the remedy."†—*Half-Yearly Abstract.*

